# Offering a lifestyle intervention to women of premenopausal age as primary prevention for cardiovascular disease? – assessing its cost-effectiveness

**DOI:** 10.1186/s12966-022-01384-z

**Published:** 2022-12-14

**Authors:** Lan Gao, Marj Moodie

**Affiliations:** Deakin Health Economics, Institute for Health Transformation, School of Health & Social Development, Faculty of Health, Deakin University, 221 Burwood Hwy, Burwood, Melbourne, Australia

**Keywords:** Lifestyle modification program, Pre-monopausal women, Cost-effectiveness, Primary prevention, Cardiovascular disease

## Abstract

**Background:**

There is limited evidence of cost-effective primary prevention interventions for cardiovascular disease (CVD) in young women. This study aimed to assess the value for money of primary prevention of CVD in this population.

**Methods:**

A Markov microsimulation model consisting of both first-ever and recurrent CVD events was developed to simulate the lifetime intervention impact on cost and health outcomes in women of premenopausal age (30 to 54 years) from the Australian healthcare system perspective. The latest wave of the Australian National Health Survey defined the modelled population’s characteristics. The intervention effectiveness of a lifestyle modification program involving changes in diet and physical activity demonstrated to be effective in this population was sourced from a systematic review and meta-analysis. The first-ever and recurrent CVD probabilities were derived from the CVD risk calculators accounting for socio-demographic and clinical characteristics. Costs and utility weights associated with CVD events and long-term management post-CVD were informed by national statistics/published literature. Sensitivity analyses were undertaken to examine the robustness of base case results.

**Results:**

The lifestyle modification program was associated with both higher costs and benefits (in terms of quality-adjusted life years, QALYs) as a primary prevention measure of CVD in premenopausal women, with an ICER of $96,377/QALY or $130,469/LY. The intervention led to fewer first-ever (N = −19) and recurrent CVD events (N = -23) per 10,000 women over the modelled life horizon. The avoided cost due to reduced hospitalisations (−$24) and management (−$164) of CVD could partially offset the cost associated with the intervention ($1560). Sensitivity analysis indicated that time horizon, starting age of the intervention, discount rate, and intervention effectiveness were the key drivers of the results. If the intervention was scaled up to the national level (N = 502,095 at-risk premenopausal women), the total intervention cost would be $794 million with $95 million in healthcare cost-savings.

**Conclusion:**

Offering a lifestyle modification program to premenopausal women in Australia as primary prevention of CVD is not cost-effective from a healthcare system perspective. We should continue to search for new or adapt/optimise existing effective and cost-effective primary prevention measures of CVD for women.

**Supplementary Information:**

The online version contains supplementary material available at 10.1186/s12966-022-01384-z.

## Background

Cardiovascular disease (CVD) persists as one of the leading causes of death among women worldwide despite substantial advances in disease awareness, prevention and treatment [1]. Managing women at risk of CVD will prevent hospital admissions, save lives and improve the quality of life of those affected. In the US, the latest statistics reported that the cost of CVD for females was U$100.3 billion in 2017–18 (43% contributed by women aged under 65, age under 55 was not reported separately) [2]. In Australia, a total of A$904 million was spent on CVD by women under 55 years in 2018–19 [3].

Age, gender, and ethnicity are non-modifiable CVD risk factors while primary prevention normally entails change of modifiable CVD risk factors to prevent the onset of CVD. Traditional CVD risk factors include obesity, hypertension, physical inactivity, and smoking, meanwhile non-traditional CVD risk factors encompassing pregnancy-related disorders, such as gestational diabetes and hypertension, preterm delivery (PTD) have also been demonstrated to contribute to the occurrence of CVD [4–6]. For example, premenopausal women are relatively protected against CVD if they do not have a history of PTD, however, women with such a history are disposed to a significantly earlier onset of CVD [4], leading to higher premature mortality and loss of productivity. In the meantime, other CVD risk factors, even though not exclusive to women, have a much higher prevalence in women than men (i.e. migraine which is associated with the risk of stroke, occurs three times more often in women) [7, 8]. The American Heart Association/American Stroke Association (AHA/ASA) guidelines for the prevention of CVD in women recommend CVD risk assessment in women with certain reproductive manifestations of CVD risk (such as pregnancy-related adverse outcomes) and suggest that female-specific risk factors may improve/complement the current CVD risk assessment strategies [9, 10]. To curb the ever-increasing disease burden of CVD, identifying effective ways to prevent CVD in specific risk groups potentially offers the best solutions; this has provoked interest to uncover interventions that are tailored to altering the CVD risk profiles of premenopausal women.

However, there is limited evidence on primary prevention designed and tested exclusively in young women. Instead, most of the primary prevention interventions have been investigated in people of older age (i.e. post-menopausal)/or both gender groups [11]. Given that there is growing appreciation that there may be gender differences in the magnitude of the relative and absolute potential benefits and risks associated with preventive interventions, identifying interventions that have proven effectiveness in young women becomes important to formulate care plans for those with increased CVD risk. Further, it is equally crucial to establish the cost-effectiveness of these effective primary preventions for CVD given constraints on the healthcare budget.

In Australia, it is recommended that the risk of CVD events is assessed using the Australian absolute cardiovascular disease risk calculator endorsed by the National Vascular Disease Prevention Alliance (NVDPA). However, this CVD risk assessment often predicts a low short-term risk for young women and prevents the opportunity to intervene early for those with non-traditional CVD risk factors [12]. A systematic review of primary prevention of CVD for young women identified that a lifestyle modification program involving changes in diet and physical activity was significantly effective in reducing the systolic blood pressure (SBP) (our meta-analysed results of five trials: −3.16 mmHg, 95%CI −6.32 to −0.01, p = 0.05, I^2^ = 0%) for women without established risk of CVD [13]. However, what is unknown is whether these reductions in SBP could be translated into long-term health benefits that resulted in the cost-effectiveness of this intervention for CVD primary prevention. Offering such a lifestyle modification to women with the increased traditional or non-traditional risk of CVD will not only serve as a way to educate women to improve their awareness of CVD (to understand the high mortality better and recognise the symptoms), but also emphasise the importance of optimal management of CVD risk factors in order to drive changes in behaviour. In this regard, assessing the cost-effectiveness of potential effective intervention in this population addresses an urgent policy-relevant question. Moreover, decision-makers increasingly require the cost-effectiveness analysis as a part of the health technology assessment to aid in the reimbursement decision-making and only interventions with demonstrated cost-effectiveness are likely to receive public subsidy. We sought to undertake a modelled economic analysis based on a Markov microsimulation using Australian national data to evaluate the cost-effectiveness of a lifestyle modification for young women as a part of a research program examining CVD primary prevention in young women.

## Methods

### Markov microsimulation structure

**Fig. 1:**
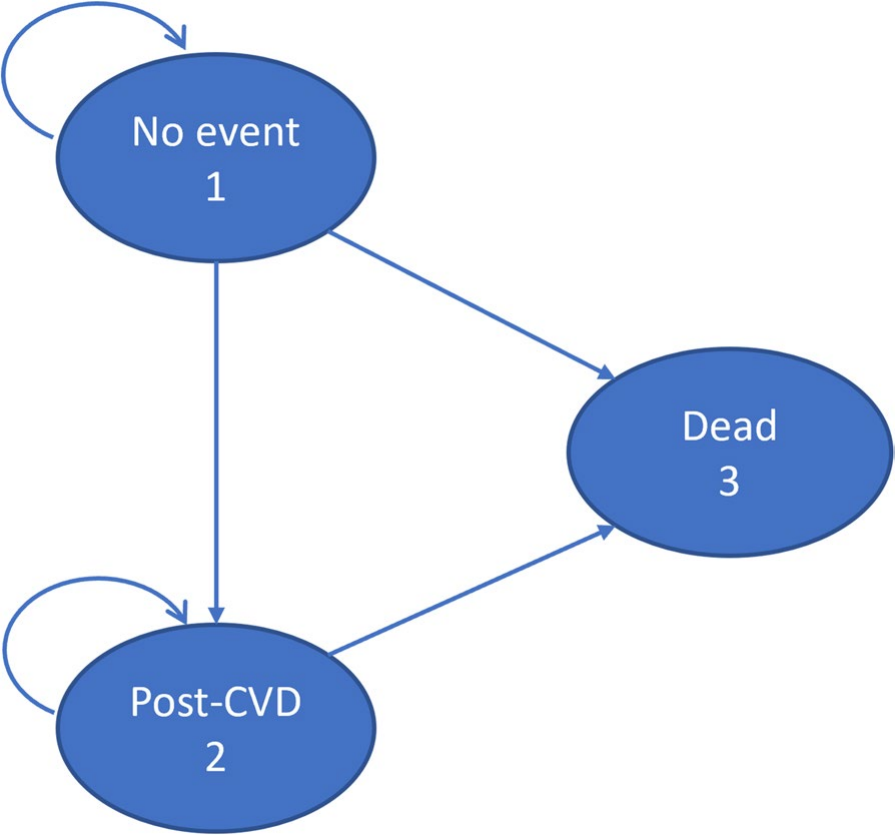
The structure of the Markov microsimulation model

A Markov microsimulation model was constructed to simulate applying the lifestyle modification program to premenopausal women in Australia. First-ever (i.e. de novo) and recurrent CVD events were considered. Upon model entry, each simulated women face the risk of a first-ever CVD or non-CVD related death. Following the first-ever CVD, they may experience another recurrent CVD or die from other causes too. The number and time of first-ever/recurrent CVD were recorded in the simulation model using the software tracker function, which informed the subsequent risk calculation of recurrent CVD. The first-ever CVD incidents included myocardial infarction (MI), unstable angina (UA), ischaemic stroke, haemorrhagic stroke, transient ischaemic attack (TIA), peripheral vascular disease (PVD), congestive heart failure (CHF), other unclassified coronary heart disease (CHD), and other CVD-related death. For women with a history of CVD, the key recurrent events were MI, stroke and vascular death. Dying from background non-CVD causes was also incorporated in the model. The structure of the health economics model is illustrated in Fig. 1.

A Markov microsimulation approach was chosen over a Markov cohort model because the former accounts for population-level heterogeneity [i.e. age, CVD risk factors (i.e. blood pressure and lipids), history of diabetes/smoking, socioeconomic status] and enables the estimation of CVD risk for individual persons. Markov microsimulation also adjusts the risk of CVD while the characteristics of individuals change as time elapses. A Markov cohort model would only allow the calculation of a single first-ever and recurrent CVD risk based on the average characteristics of the cohort (i.e. the means of age and other CVD risk factors) and does not allow the memory of the prior event (e.g. the history of CVD increases the risk the recurrent events). In contrast, the Markov microsimulation model estimates the CVD risk on an individual basis that addresses the varied risk according to different levels of blood pressure, age, lipids, socioeconomic status, etc..

### Intervention effect

The intervention effect of lifestyle modification program involving the changes in diet and/or physical activity was derived from a systematic literature review of primary prevention for CVD in young women conducted by our group [13–19]. As the systematic review and meta-analysis of primary prevention intervention of CVD in young women only identified one single invention as being effective in reducing the CVD risk in the target population, this intervention was subsequently chosen in the current cost-effectiveness analysis. Basically, the program entailed promotion of physical activity and/or healthy diet using varied strategies. The meta-analysed results from five randomised controlled trials were: −3.16 mmHg, 95%CI −6.32 to −0.01, p = 0.05, I^2^ = 0% for women without established risk of CVD [13–19], which was borderline significant. It was assumed that this program would be offered to eligible women (premenopausal with CVD risk factors) for five-year (i.e. repeated annually for five times) and the interventional effect was sustained over this period only. After five years, there was no life modification program. In addition, it was assumed that women stopped receiving lifestyle modification programs once the de novo CVD event occurred. The intervention effect (i.e. lowered SBP) was reflected in the simulation model by the reduced risk calculation of the first-ever CVD using the PREDICT risk calculator and, subsequently the risk of recurrent CVD while holding the other model inputs identical.

### Event probability

#### First-ever CVD

The probability of first-ever CVD events was derived from the PREDICT-I CVD risk prediction model [20]. PREDICTis an ongoing cohort study in New Zealand that recruits participants (44% of female participants and 66.8% under the age of 55 years) when primary care providers complete standardised CVD risk assessments using PREDICT decision support software. The software auto-populates a PREDICT risk factor template from patient records while clinicians are required to fill in any missing fields. The medical record of each participant is automatically and regularly linked to national databases registering drug dispensing, hospitalisations, and deaths attributable to CVD. The first version of the CVD risk prediction model was developed based on a population consisting of 452,092 males and females aged from 30 to 74 years between 2002 and 2015 [20]. The CVD risk prediction model estimates the probability of a de novo CVD event over five-years. The population composition is highly comparable between New Zealand and Australia. The median age was 47 in Australia vs 48 in NZ, with around a 7.0% of death rate per 1000 population and similar ethnic group distributions [21].

As the health economics model adopted a yearly Markov cycle, the probability of de novo CVD over the next five-years was converted to a yearly probability by utilising the TreeAge built-in function (probtoprob, to estimate the yearly event probability from five-year event probability). The probability of each subtype of CVD (fatal or non-fatal), including MI, UA, stroke (ischaemic or haemorrhagic), TIA, PVD, CHF, unclassified CHD, and other CVD-related death was derived from the paper detailing the PREDICT-I risk calculator [20]. The details for the first-ever CVD risk prediction model and probabilities of each subtype of CVD are summarised in Supplementary Tables 1 and 2.

#### Recurrent CVD

The probability of recurrent CVD was calculated based on SMART risk scores [22], which estimatethe 10-year risk for MI, stroke or vascular death in patients with previous CVD. The SMART risk score was originated from an ongoing cohort study in the Netherlands that contains 5788 patients (aged between 53 and 68 years) with clinical manifestations of cerebrovascular disease (TIA, stroke), coronary artery disease (including MI and coronary revascularisation), PVD and abdominal aortic aneurysm [22]. Similar to the first-ever CVD, the TreeAge built-in function (probtoprob) was employed to estimate the yearly event probability from the 10-year event probability. The beta coefficients for the SMART risk prediction model are shown in Supplementary Table 3.

Based on the characteristics of the simulated women, first-ever and recurrent CVD were calculated using the relevant risk predictors for females.

#### Background non-CVD mortality

All-cause mortality data from the Australia Bureau of Statistics (ABS) was sourced to estimate the background non-CVD mortality. In particular, CVD-related mortality by age and gender group was subtracted from the all-cause mortality to approximate the non-CVD mortality. Only the statistics for females were used for this transition probability. The non-CVD mortality is provided in Supplementary Table 4.

### Population

Premenopausal women (aged between 30 and 54 years) were simulated, based on population statistics from the Australian Bureau of Statistics (ABSa). Baseline socio-demographic (age, weight, height, socioeconomic status) and health-related (blood pressure, smoking status/history, blood lipids (total cholesterol, TC and high-density lipoprotein, HDL), diabetes history characteristics by age and gender were defined according to the latest wave of Australia Health Survey.

#### Baseline characteristics

The socio-demographic characteristics including age (i.e. the distribution across age bands), index of relative socioeconomic disadvantage (i.e. distribution from first to the fifth quintile), smoking status/history (i.e. the distribution by age band), diabetes status (i.e. prevalence of diabetes by age group), level of systolic blood pressure (SBP) (i.e. distribution of SBP from <100 mmHg to >170 mmHg by age band), and level of blood lipid (including TC and HDL, in mmol/L) (i.e. distribution of blood lipids by age) were sourced from the Australia Health Survey. The details of each baseline characteristic are presented in Supplementary Tables 5 to 10.

#### Changes in systolic blood pressure and lipids

Given the trend in the change of blood pressure and lipids with aging, the variation in these two parameters by age was sourced from population-level cohort studies [23, 24]. In the Markov microsimulation model, each modelled individual had the systolic blood pressure and lipids levels (TC and HDL) adjusted according to their age during the simulation.

#### Disease history

CVD history (i.e. MI, stroke, PVD, etc.) and time point (which was used to calculate the time since the first CVD event) of the corresponding event of each individual were documented in the model. These CVD histories automatically updated in the record of each modelled patient and subsequently impacted the probability of subsequent recurrent CVD events (i.e. patients with histories of CVD have higher risk of experiencing a subsequent CVD event) according to the risk equation for the recurrent CVD.

### Utility weights

The utility weight of the general female Australian population by age was derived from an Australian study [25]. For people who experienced an ischaemic stroke, post-stroke utility was calculated as the weighted average according to modified Rankin scale (mRS) score (i.e. from mRS 0 to mRS 6 weighted by the distribution of mRS score). The utility weights associated with post-haemorrhagic stroke, post-MI, post-CHF, and post-PVD were sourced from published literature, while the utility weight for post-other CHD was calculated as the arithmetic mean of utility scores across post-stroke, post-MI, post-CHF, and post-PVD.

### Costs

An Australian healthcare system perspective was taken to measure the costs borne by the healthcare system. Costs related to hospitalisation due to the acute CVD events, long-term disease management and intervention (i.e. lifestyle modification program) were incorporated into the cost-effectiveness analysis. The costs associated with acute hospitalisation were generated from the government administrative databases while the CVD management costs were sourced from published literature. Costs were expressed in Australian dollarsfor the 2018 reference year.

### Cost of intervention

The lifestyle intervention generally included physical activity promotion, diet and lifestyle education/counselling, and multiple follow-ups over time. Since this economic evaluation is based on modelling without primary data collection, the cost of providing a lifestyle modification program was calculated based on a previous study [26], and included the following components:

- group-based (6 people) physical activity training session three times per week (Medical Benefits Schedule [MBS] 10,953, $63.25 × 3/8 = $31.63/week)
- three individualised dietary counselling sessions for the first three months (MBS item no. 10954, $63.25 × 3 = $181.80 and followed by regular group meetings for anotherthree months; (MBS 81105, $20.20/session×3 = $60.60)
- weekly texts reminder over 6 months ($1/person/week)

The unit cost of physical activity training session and diet counselling were sourced from MBS (i.e. allied health practitioner). The cost for the weekly text reminders was estimated based on an intervention with a similar component [27]. The total cost of lifestyle modification program was $335.63 per participant.

Utility weight and costs are summarised in Table 1 [25, 28–36]].

**Table 1:** Key model inputs for the cost-effectiveness analysis

Variable	Definition	Basecase value	Reference	Sensitivity analysis	
		Female		DSA	PSA^**a**^
**Utility**					
u_norm	Age 16–19	0.87	Hawthorne et al. 2013 [25]		0.17
	Age 20–29	0.84			0.2
	Age 30–39	0.84			0.21
	Age 40–49	0.81			0.22
	Age 50–59	0.8			0.23
	Age 60–69	0.79			0.22
	Age 70–79	0.76			0.24
	Age 80–85	0.68			0.26
u_stroke_IS				0–1	
	mRS 0	0.63	Sturm et al. 2002 [28]		
	mRS 1	0.63			
	mRS 2	0.4			
	mRS 3	0.18			
	mRS 4	0.06			
	mRS 5	0.02			
u_TIA	Utility value post TIA	0.63	Sturm et al. 2002 [28]		
u_stroke_HE	Utility value post a haemorrhagic stroke	0.3	Shin et al. 1997 [29]	0–0.6	
u_MI	Utility value post a MI	0.87	Tsevat et al. 1993 [30]	0.80–0.95	
u_CHF	Utility value post CHF	0.67	Pandor et al. 2013 [31]		
u_PVD	Utility value post PVD	0.79	Itoga et al. 2018 [32]		
u_otherCHD^b^	Utility value post other CHD	0.665	Calculated		
u_postCVD	Utility value post CVD	0.626			
**Costs**					
**Acute phase**					
c_MI	cost of hospitalisation for a myocardial infarction	$ 8944	NHCDC 2015–16 cost report		894
c_UA	cost of hospitalisation for an unstable angina	$ 6077	NHCDC 2015–16 cost report		608
c_otherCVD	cost of hospitalisation for other CVD	$ 9796	NHCDC 2015–16 cost report		980
c_stroke_IS	cost of hospitalisation for an ischaemic stroke	$ 10,712	NHCDC 2015–16 cost report	5733-22,245	1071
c_stroke_HA	cost of hospitalisation for a haemorrhagic stroke	$ 15,563	NHCDC 2015–16 cost report	6116-22,245	1556
c_TIA	cost of hospitalisation for a TIA	$ 5172	NHCDC 2015–16 cost report	3339-7005	
c_PVD	cost of hospitalisation for a peripheral vascular disease	$ 6241	NHCDC 2015–16 cost report	–	
c_CHD	cost of hospitalisation for a congestive heart failure	$ 7003	NHCDC 2015–16 cost report	3037-12,423	700
c_CVD_death_other	cost of other CVD-related death	$ 8688	average		
**Long-term management**					
c_mgmt_MI	cost of management post MI	$ 4302	Turkstra et al. 2013 [33]		430
c_mgmt_UA	cost of management post UA	$ 4302			
c_mgmt_otherCVD	cost of management post otherCVD	$ 4667	average		
c_mgmt_stroke	cost of management post stroke	$ 6410	NHCDC 2015–16 cost report; Cobiac et al. 2012 [34]		641
c_mgmt_TIA	cost of management post TIA	$ 1431	mRS0		
c_mgmt_PVD	cost of management post PVD	$ 5062	Itoga et al. 2018 [32]		
	mild case (intermittent claudication), outpatient visit every per 3 months	$ 150	MBS 23		
	severe case (critical limb ischaemia), outpatient visit every month, 30 min nurse time every 2 weeks	$ 1990	MBS 104 & MBS 82210		
	amputated case (GBP 23,502/AUD 41902 per year)	$ 41,902			
c_mgmt_CHD	cost of management post CHD	$ 6494	Maru et al. 2018 [35]		649
c_mgmt_postCVD	the average cost of post CVD management	$ 4667	Assumption		
c_intervention	group physical activity training	$ 31.63	MBS 10953		
	individual dietitian	$ 60.60	MBS 10954		
	group dietitian	$ 80.80	MBS 81105		
	text reminder	$ 1.00	Gao et al. 2018 [36]		

^a^SD and the mean from the base case value were used to define beta distribution for utility variables or gamma distribution for cost variables in the probabilistic sensitivity analyses, according to the empirical evidence

^b^average across stroke, MI, CHF, and PVD

### Cost-effectiveness analysis

Quality-adjusted life year (QALY) was the primary outcome measure for the cost-effectiveness analysis. Life years (LY) was the secondary outcome measure. The incremental cost-effectiveness ratio (ICER) was calculated for both primary and secondary outcome measures. All costs and benefits were discounted at a rate of 3% per annum. Half-cycle correction was applied assuming all the events occurred during the middle of the Markov cycle (i.e. per year). The total number of CVD events over the modelled time horizon was estimated by summing the number of CVD events across all simulated individuals and was presented by CVD subtype. The often quoted willingness-to-pay (WTP) per QALY of A$50,000 in Australia was adopted to determine the cost-effectiveness of the evaluated intervention [37].

### Subgroup analysis

In order to provide further suggestions for public funding decision-making, three subgroup analyses were undertaken to examine the cost-effectiveness of lifestyle modification programs offered to women aged from 30 to 40, 40 to 50, or 50 to 55 years, by reconfiguring the age distribution of the modelled population.

### Sensitivity analysis

Deterministic sensitivity analysis (DSA) which varied parameters within a plausible range was performed to examine which parameter impacted on the base case ICER and the extent of such impact. The extreme value analysis was performed to assess the impact of 0% adherence to the intervention after one year. Probabilistic sensitivity analyse (PSA) by incorporating the distribution of key uncertain parameters (i.e. cost and utility weights) was carried out to further explore the robustness of the base case results (Table 1). An incremental cost-effectiveness plane and acceptability curve were presented to illustrate the results from PSA.

### Budget impact analysis

The total cost to the healthcare system if the lifestyle modification program was implemented Australia-wide for the next 5-years was estimated. Using the population size derived from the ABS, the total budget of this CVD primary prevention program for young women was calculated by multiplying the per capita cost by the total number of eligible female Australians. In order to estimate the total population, it was assumed that premenopausal women with histories of gestational hypertension, diabetes mellitus, preterm delivery or polycystic ovary syndrome would be eligible to receive such an intervention. The prevalence of these conditions was derived from national statistics in Australia [38–40].

## Results

### Cost-effectiveness analysis

The base care result showed that lifestyle modification program was associated with higher costs and marginally increased benefits (QALY and LY). The average lifetime cost due to CVD and QALYs for women aged between 30–54 years were $8133 and 18.07 (lifestyle modification program) vs $6743 and 18.06 (usual care), respectively. The corresponding ICER was $108,801/QALY (Table 2).

**Table 2:** Results of base case cost-effectiveness analysis

	Lifestyle modification	Usual care	Difference	ICER
**Total cost**	$8133	$6743	$1389	–
**QALY**	18.070	18.057	0.0128	$108,801/QALY
**LY**	23.084	23.074	0.0094	$147,941/LY
Cost of intervention	$1560	$0	$1560	
Cost of CVD-related hospitalisation	$904	$928	-$24	
Cost of CVD management	$5568	$5732	-$164	
**Number of first ever CVD events**^**a**^	1000	1019	-19	
Myocardial infarction	339	346	-7	
Unstable angina	154	156	-2	
Ischaemic stroke	147	150	−3	
Haemorrhagic stroke	42	43	−1	
Transient ischaemic attack	75	77	−2	
PVD	56	56	0	
Congestive heart failure	117	120	−3	
Other CHD	49	50	−1	
Other CVD death	21	21	0	
**Number of recurrent CVD events**	711	734	−23	
Nonfatal stroke	182	187	−5	
Nonfatal myocardial infarction	209	219	−10	
CVD death	320	328	−8	

*QALY* quality-adjusted life year, *LY* life year, *ICER* incremental cost-effectiveness ratio, *CVD* cardiovascular disease, *CHD* coronary heart disease, *PVD* peripheral vascular disease

^a^per 10,000 patients

The total costs of the intervention ($1560) were partially offset by the savings in CVD-related hospitalisations (−$24) and CVD disease management (−$164) over the modelled time horizon.

### Number of CVD simulated

**Fig. 2:**
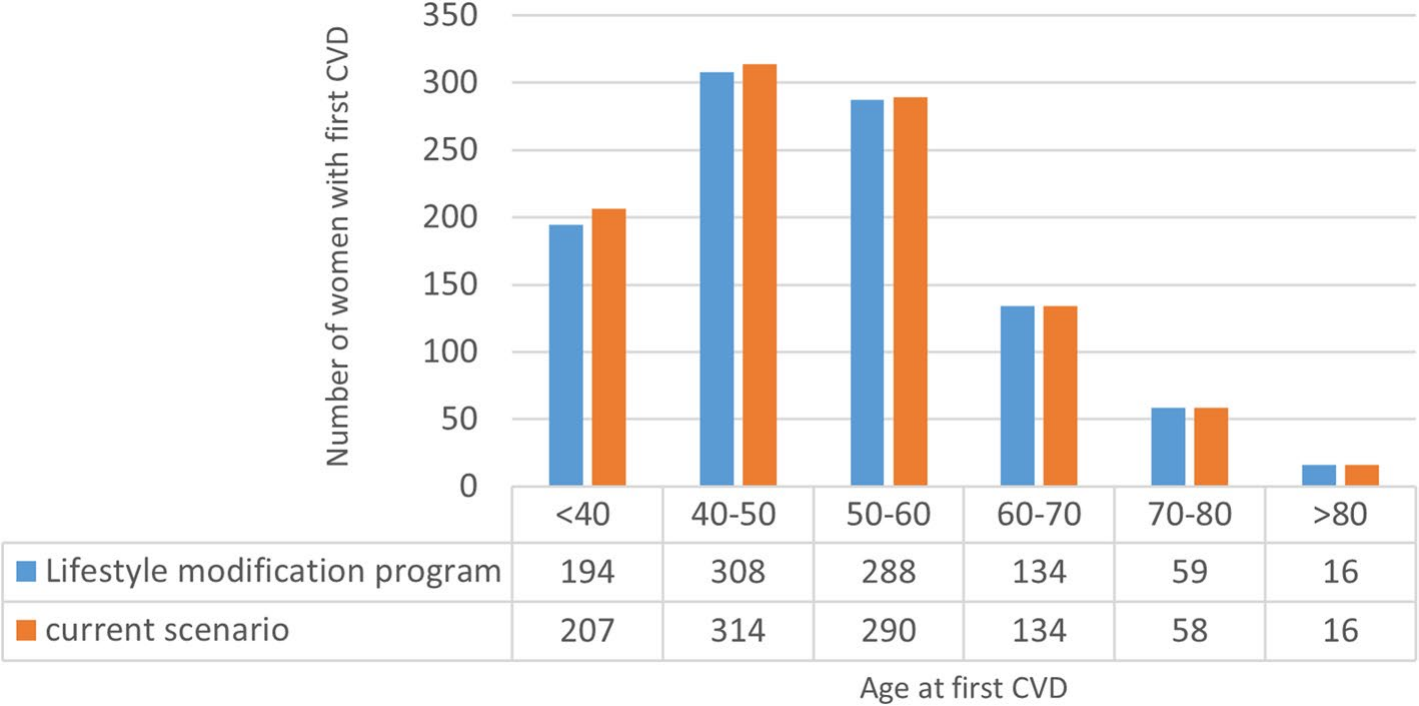
Number of the first CVD by age groups

The total number of first-ever CVD and recurrent CVD events was 1000 and 711 (lifestyle modification group) vs 1019 and 734 (usual care) per 10,000 women simulated. MI, UA, and CHF were the top three CVD events that occurred most frequently as the de novo event whereas CVD-related death was the most prominent recurrent event for patients with an established history of CVD. The average age for first-ever CVD was 50.5 years in the lifestyle modification group and 50.1 years in the usual care group (i.e. the intervention delayed the time of the first CVD by 0.4 year across all the simulated population). Since the modelled population only consisted of women aged from 30 to 54, more first-ever CVD events occurred in the 40 to 50 years age band, while more de novo CVD (N = 13) was avoided in the younger than 40 age group, indicating a beneficial impact of early prevention of CVD. The distribution of first-ever CVD across age-band by groups is shown in Fig. 2. Primary prevention of CVD also contributed to fewer recurrent CVD events (23 less per 10,000 women) owing to the increased risk of recurrent CVD after the first event.

### Subgroup analysis

Subgroup analysis conducted by varying the target age group of premenopausal women suggested that the lifestyle modification program has a better chance of being cost-effective if geared toward young women. The highest ICER was observed in the highest age group for premenopausal women, highlighting the importance of early preventive intervention (Table 3).

**Table 3:** Results of subgroup analysis

	Lifestyle modification	Usual care	Difference	ICER
***Age from 30 to 40***				
Total cost	$10,691	$9434	$1258	–
QALY	19.060	19.042	0.0187	$67,298/QALY
LY	24.023	24.012	0.011	$115,736/LY
Cost of intervention	$1555	$0	$1555	
Cost of CVD-related hospitalisation	$1143	$1176	-$33	
Cost of CVD management	$7993	$8258	-$265	
***Age from 40 to 50***				
Total cost	$6818	$5401	$1417	
QALY	17.826	17.817	0.0093	$152,003/QALY
LY	22.917	21.910	0.007	$211,555/LY
Cost of intervention	$1562	0	$1562	
Cost of CVD-related hospitalisation	$793	$813	-$20	
Cost of CVD management	$4462	$4587	-$125	
***Age from 50 to 54***				
Total cost	$4574	$3061	$1513	–
QALY	16.394	16.390	0.004	$372,597/QALY
LY	21.357	21.354	0.003	$432,417/LY
Cost of intervention	$1567	$0	$1567	
Cost of CVD-related hospitalisation	$537	$545	-$8	
Cost of CVD management	$2471	$2516	-$45	

### Sensitivity analysis

**Fig. 3:**
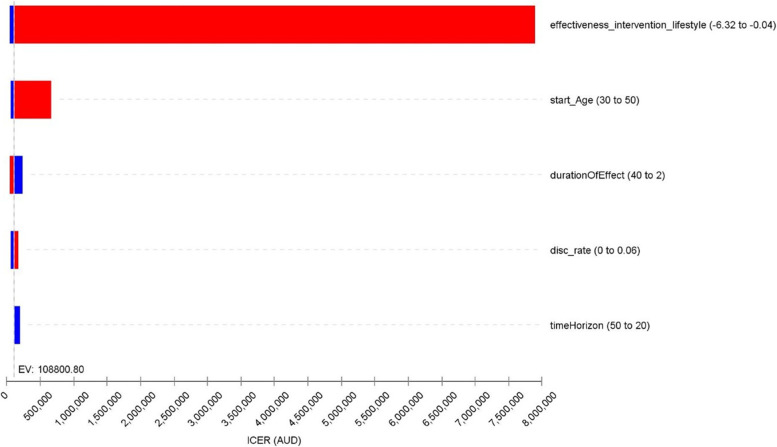
Tornado diagram for the one-way sensitivity analyse

**Fig. 4:**
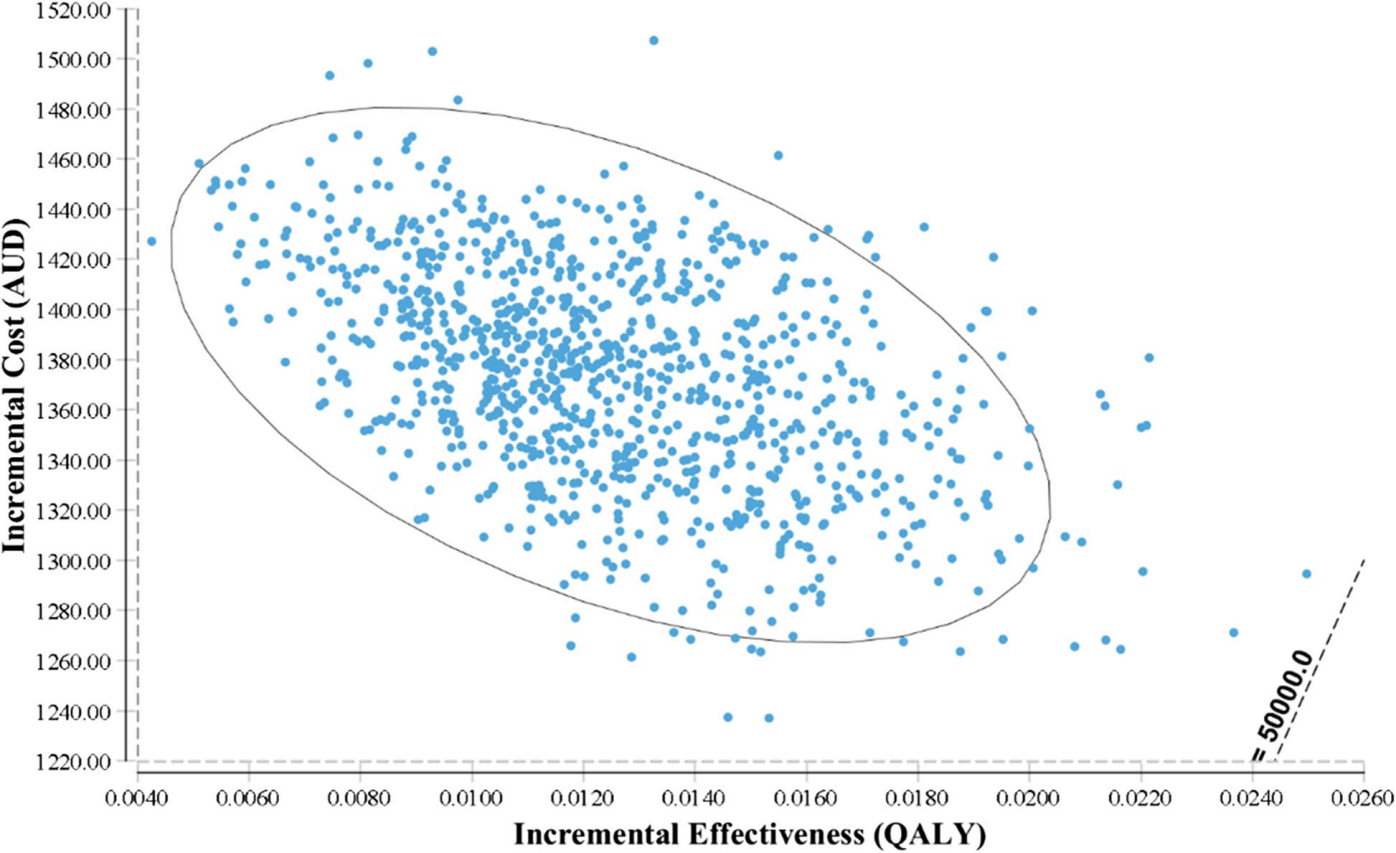
Cost-effectiveness plane from the probabilistic sensitivity analysis. *The intervention has a 100% probability being cost-ineffective

One-way deterministic sensitivity analysis indicated that the time horizon, starting age of the intervention, discount rate, effectiveness of the intervention, and duration of the intervention effect were key determinants of the cost-effectiveness of such intervention, driving substantial changes in the ICER (Fig. 3-A). Meanwhile, utility weights of post-MI, ischaemic/haemorrhagic stroke, CHF, and costs of acute hospitalisation due to ischaemic stroke and CHD impacted the ICER to a much lesser extent (Fig. 3-B). The extreme value analysis suggested that 0% adherence to the program was associated with lower health benefits and reduced incremental cost (due to lowered intervention costs), leading to decreased ICER (Supplementary Table 11). The probabilistic sensitivity analysis suggested that the intervention is unlikely to be cost-effective in its current form (Fig. 4).

### Budget impact analysis

The total female population that was eligible for the lifestyle modification program was around 502,095 in 2017. With a per capita intervention cost of $335.63, the total intervention cost would be $794 million over 5-years while the cost-offset due to acute hospitalisation and long-term management cost related to CVD onset was $95 million over the lifetime of this baseline population.

## Discussion

From this modelled study, we observed that the lifestyle intervention, which was associated with higher costs and benefits (i.e. QALY and LY gains) offered to women of premenopausal age was not cost-effective from an Australian healthcare system perspective. Meanwhile, it could potentially prevent 7448 premenopausal women in Australia (total population was 4,184,123 in 2017) from experiencing a de novo CVD event and save hospitalisation and management costs due to the occurrence of CVD. In particular, it led to avoidance of 7 MI, 2 UA, 4 strokes, 2 TIAs, and 3 CHF per 10,000 women intervened. However, the cost offsets from the avoided hospitalisation and long-term management did not counterbalance the relatively high costs of intervention implementation. Pharmacological interventions that act to lower blood pressure like thiazide diuretic, calcium channel blocker, ACE inhibitor all cost less than the lifestyle modification program assessed in this economic evaluation, and since they generate greater health benefits in reducing systolic blood pressure, they are generally highly cost-effective.

Threshold analysis was also undertaken to determine a potential strategy to make this intervention cost-effective. The key drivers of the intervention’s cost-effectiveness outcome included intervention effectiveness, duration of intervention effectiveness, and discount rate. If the intervention effect could be sustained for 20 years, this lifestyle modification program would become cost-effective, while in the base case, the intervention effect was assumed to be sustained for five-year only to be conservative. The studies around non-pharmacological primary prevention of CVD have normally adopted a pragmatic design that employs the intermediate endpoint (i.e. increase in physical activity, decrease in blood pressure or lipids) rather than final health outcomes (i.e. mortality or occurrence of CVD event), to avoid the necessity to follow up patients in the long term. The longest follow-up in those studies was around one year [41]. Without observing the eventual CVD/mortality events, this study translated an intermediate reduction in SBP into the probability of first-ever CVD using a CVD risk calculator based on a large cohort study in New Zealand [20]. It is believed that this approach reflected the heterogeneity in the general population simulated and also properly captured the benefits in terms of risk modifications (i.e. lowered systolic blood pressure). In addition, lifestyle modification was also observed to have a positive impact on the levels of fasting blood glycaemia and physical activity, however, these incidental benefits are not considered in the current model; this may lead to underestimation of the overall intervention effect. In addition, productivity benefits from avoiding future CVD events were not considered in the long-term model.

Heart disease is a leading killer of women in Australia, with the total number of deaths exceeding that related to breast cancer and diabetes combined [42, 43]. It is also concerning that women had low awareness of heart disease, leading to often poorer health outcomes than for men [44, 45]. Women are also less likely to have heart health checks (~30%), and consult with a health professional about the risk factors of CVD [45]. On the contrary, women possess gender-unique risk factors that are highly associated with CVD. For example, histories of gestational diabetes or pre-eclampsia, PTD, polycystic ovary syndrome, and early menopause all contribute to a significantly increased risk of CVD.

Primary interventions for CVD are abundant, involving a wide range of initiatives from pharmaceuticals to lifestyle modification programs. These studies have generally recruited people of older age (i.e. post-menopausal) and/or both gender groups [46–48]. However, effective intervention targeting women (especially young women) is scarce. There is a growing appreciation that there may be gender differences in the magnitude of the relative and absolute potential benefits and risks associated with preventive interventions [49, 50]. Hence, evidence from studies in unrestricted populations is not necessarily applicable to women of younger age (i.e. premenopausal). Besides, no specific study has been designed to investigate how to improve the CVD risk profiles for women with histories of pregnancy-related complications (i.e. PTD, gestational diabetes). Subsequently, only interventions that have shown promise in premenopausal women were evaluated in this modelled economic evaluation.

Existing economic evaluation around lifestyle modification, which includes diet and/or physical activity-based components for the primary prevention of CVD, almost consistently showed it was not cost-effective. Australian studies generally report that lifestyle programs involving physical activity and/or dietary advice offered to people with varied absolute risk of CVD was not cost-effective, with the corresponding ICER per disability-adjusted life year (DALY) ranging from $79,000 [51] to $1,400,000 [26]. Of note, a lifestyle modification program incorporating a physical activity component consistently yielded a better cost-effectiveness outcome (ICER ranged from $79,000 to $130,000/DALY) [51, 52] than a program only concerned about diet optimisation (ICER was between $160,000 to $1 M/DALY) [26, 53, 54]. The ICER from the current study fell well within the range of the published economic evaluation in this regard. The difference in the ICER between studies could be explained by the fundamental difference in the modelling technique (Markov cohort vs Markov microsimulation) and, most importantly, the intervention effectiveness. However, it is believed that a microsimulation that accounts for individual heterogeneity (i.e. blood pressure, lipid profile, smoking and diabetes histories, etc.) is more appropriate to model the interventions for CVD. Given prior economic analyses of lifestyle modification intervention unanimously targeted at people with traditional CVD risk factors and more advanced age, our presented study will add to the existing literature in regard to the cost-effectiveness of primary prevention intervention of CVD in young women with non-traditional CVD risk factors.

This study is the first attempt to evaluate the cost-effectiveness of a lifestyle modification program that is likely to be effective in women of premenopausal age. The economic evaluation was based on a population-level CVD risk calculator that predicts the first-ever and recurrent CVD event over the lifetime. The characteristics of the general Australian population including socio-demographic, and CVD risk factors were derived from the latest National Health survey data to ensure the generalisability of the cost-effectiveness conclusion. This modelling technique overcomes the limitation of a Markov cohort model which is their memoryless for patient’s history. However, this study comes with limitations. First, intervention effectiveness was derived from a meta-analysis of studies that only followed up patients for the short-term. There is uncertainty about the sustainability of long-term intervention effectiveness. In the base case, it was assumed that the intervention would be offered every year for a five-year period, and there would be no intervention effect left afterwards. Additionally, the shorter duration of intervention effectiveness was examined in the sensitivity analysis. Second, the risk calculator for recurrent CVD was derived from a cohort with ages ranging from 52 to 68 years whereas our modelled population had a youngest age of 30 years. But it is noted that only a small proportion (19.4%) of modelled women had first ever CVD younger than 40 years. Thus, the prediction for the recurrence for a majority of the modelled population was legitimate. Third, only the recurrent stroke and MI were modelled following the de novo CVD due to the insufficient evidence to inform the model. Since the intervention effectiveness was only applied to women without a history of CVD, the post-CVD trajectory would be similar between the two scenarios, and this is unlikely to have biased the results. Lastly, the baseline CVD biomarkers, including TC and HDL were sourced from the 2011–12 National Health Survey. This may not be the true reflection of the contemporary lipids profile of Australian women. However, since it was applied to simulated women in both scenarios, the impact on the cost-effective conclusion is likely to be very minimal.

## Conclusions

Offering lifestyle modification program to premenopausal women in Australia as the primary prevention of CVD is not cost-effective from a healthcare system perspective due to the high intervention cost and uncertainty in the sustainability of intervention effectiveness. However, it is important to educate premenopausal women, especially those with traditional and non-traditional CVD risk factors about the high mortality of CVD in women and highlight the importance of risk factor management. We should continue to search for new or adapt/optimise existing effective and cost-effective primary prevention of CVD strategies for women.

## Supplementary Information

**Additional file 1: Supplementary Table 1.** Beta-coefficient for estimating the probability of first-ever CVD event. **Supplementary Table 2.** Details of each type of CVD_ first ever CVD. **Supplementary Table 3.** Beta-coefficient for estimating the probability of recurrent CVD event. **Supplementary Table 4.** Non-CVD related background mortality. **Supplementary Table 5.** Australia female population size. **Supplementary Table 6.** Index of Relative Socioeconomic Disadvantage. **Supplementary Table 7.** History of smoking and diabetes. **Supplementary Table 8.** Level of systolic blood pressure by age. **Supplementary Table 9.** Systolic blood pressure increases by age. **Supplementary Table 10.** Level of total cholesterol and high-density lipid by age. **Supplementary Table 11.** Extreme value analysis by assuming a 0% adherence rate after one year of the intervention.

## Data Availability

Data sharing is not applicable to this article as no datasets were generated or analysed during the current study.

## Abbreviations

CVD: Cardiovascular disease
CHD: Coronary heart disease
PVD: Peripheral vascular disease
ICER: Incremental cost-effectiveness ratio
QALY: Quality-adjusted life year
LY: Life year
ABS: Australian Bureau of Statistics
WTP: willingness-to-pay
PTD: Preterm delivery
SBP: Systolic blood pressure
