# Supplementary material for: Offering a lifestyle intervention to women of premenopausal age as primary prevention for cardiovascular disease? – assessing its cost-effectiveness
Source: Int J Behav Nutr Phys Act. 2022 Dec 14;19:152. doi: 10.1186/s12966-022-01384-z (PMC9749355; doi:10.1186/s12966-022-01384-z)
Supplement: Supplementary file 1 — Additional file 1: Supplementary Table 1. Beta-coefficient for estimating the probability of first-ever CVD event. Supplementary Table 2. Details of each type of CVD_ first ever CVD. Supplementary Table 3. Beta-coefficient for estimating the probability of recurrent CVD event. Supplementary Table 4. Non-CVD related background mortality. Supplementary Table 5. Australia female population size. Supplementary Table 6. Index of Relative Socioeconomic Disadvantage. Supplementary Table 7. History of smoking and diabetes. Supplementary Table 8. Level of systolic blood pressure by age. Supplementary Table 9. Systolic blood pressure increases by age. Supplementary Table 10. Level of total cholesterol and high-density lipid by age. Supplementary Table 11. Extreme value analysis by assuming a 0% adherence rate after one year of the intervention. [file 12966_2022_1384_MOESM1_ESM.docx]

# Online supplementary material

**Supplementary Table 1 Beta-coefficient for estimating the probability of first-ever CVD event**

| **Variable** | **Description** | **Value** |
| --- | --- | --- |
| coefficient_age | beta-coefficient from the PREDICT-II equation | 0.0756412 |
| coefficient_SEIFA | beta-coefficient from the PREDICT-II equation | 0.1080795 |
| coefficient_smoker_ex | beta-coefficient from the PREDICT-II equation | 0.087476 |
|  |  |  |
| coefficient_diabetes | beta-coefficient from the PREDICT-II equation | 0.5447632 |
| coefficient_SBP | beta-coefficient from the PREDICT-II equation | 0.0136606 |
| coefficient_TCHDL | beta-coefficient from the PREDICT-II equation | 0.1226753 |
| coefficient_OBPLM | beta-coefficient from the PREDICT-II equation | 0.339925 |
| coefficient_age_diabetes | beta-coefficient from the PREDICT-II equation | -0.0222549 |
| coefficient_age_SBP | beta-coefficient from the PREDICT-II equation | -0.0004425 |
| coefficient_OBPLM_SBP | beta-coefficient from the PREDICT-II equation | -0.004313 |
|  |  |  |
| age_centred | centred value for age | 55 |
| SEIFA_centred | centred value for SEIFA | 3 |
| SBP_centred | centred value for SBP | 135 |
| TC_HDL_centred | centred value for TC/HDL ratio | 5 |

TC HDL: total cholesterol and high-density lipoprotein; SBP: systolic blood pressure; OBPLM: oral blood pressure lowering medication; SEIFA: socio-economic Index for Areas.

**Supplementary Table 2 Details of each type of CVD_ first ever CVD**

| **Outcome type** | **Non-Fatal events, n** | **Fatal events, n^a^** | **Proportion of all CVD (%)** |
| --- | --- | --- | --- |
| Myocardial infarction | 4,984 | 188 | 0.34 |
| Unstable angina | 2,275 | 11 | 0.15 |
| Other coronary heart disease | 343 | 436 | 0.05 |
| Ischaemic stroke | 2,124 | 156 | 0.15 |
| Haemorrhagic stroke | 445 | 205 | 0.04 |
| Transient ischemic attack | 1,123 | 0 | 0.07 |
| Peripheral vascular disease | 790 | 62 | 0.06 |
| Congestive heart failure | 1,795 | 113 | 0.12 |
| Other Ischaemic CVD-related deaths | n/a | 336 | 0.02 |
| **Total CVD events (N = 15,386)^b^** | **13,879** | **1,507** | **100** |

^a^ If a participant died within 28 days of a non-fatal CVD event, the event was counted as fatal; ^b^ If a participant had more than one type of CVD event, only the first was counted.

Reference: Pylypchuk R, Wells S, Kerr A, Poppe K, Riddell T, Harwood M, et al. Cardiovascular disease risk prediction equations in 400 000 primary care patients in New Zealand: a derivation and validation study. Lancet (London, England). 2018 May 12;391(10133):1897-907.

**Supplementary Table 3 Beta-coefficient for estimating the probability of recurrent CVD event**

| **Variable** | **Description** | **Value** |
| --- | --- | --- |
| coefficient2_age | beta-coefficient from the SMART risk score | -0.085 |
| coefficient2_age2 | beta-coefficient from the SMART risk score | 0.00105 |
| coefficient2_diabetes | beta-coefficient from the SMART risk score | 0.223 |
| coefficient2_smoke_current | beta-coefficient from the SMART risk score | 0.262 |
| coefficient2_SBP | beta-coefficient from the SMART risk score | 0.00429 |
| coefficient2_TC | beta-coefficient from the SMART risk score | 0.0959 |
| coefficient2_HDL | beta-coefficient from the SMART risk score | -0.426 |
| coefficient2_time_firstCVD | beta-coefficient from the SMART risk score | 0.0229 |
| coefficient2_stroke_history | beta-coefficient from the SMART risk score | 0.406 |
| coefficient2_CHD_history | beta-coefficient from the SMART risk score | 0.14 |
| coefficient2_PVD_history | beta-coefficient from the SMART risk score | 0.2832 |
| coefficient2_egfr | beta-coefficient from the SMART risk score | -0.0532 |
| coefficient2_egfr2 | beta-coefficient from the SMART risk score | 0.000306 |
| coefficient2_hsCRP | beta-coefficient from the SMART risk score | 0.139 |

TC: total cholesterol; HDL: high-density lipoprotein; SBP: systolic blood pressure; OBPLM: oral blood pressure lowering medication; PVD: peripheral vascular disease; egfr: estimated glomerular filtration rate; hsCRP: high-sensitivity C-reactive protein

**Supplementary Table 4 Non-CVD related background mortality**

| **Age** | **Non-CVD death rate** |
| --- | --- |
| 18 | 0.000202 |
| 25 | 0.000312 |
| 35 | 0.000677 |
| 45 | 0.001545 |
| 55 | 0.003318 |
| 65 | 0.008087 |
| 75 | 0.024712 |
| 85 | 0.085399 |
| 95 | 0.212265 |

Reference: 3303.0 Causes of Death, Australia 2017

**Supplementary Table 5 Australia female population size**

| Age (years) | **New South Wales** | **Victoria** | **Queensland** | **South Australia** | **Western Australia** | **Tasmania** | **Northern Territory** | **Australian Capital Territory** | **Australia** |
| --- | --- | --- | --- | --- | --- | --- | --- | --- | --- |
| 18 | 46,410 | 37,519 | 30,091 | 10,371 | 15,268 | 3,213 | 1,378 | 2,485 | 146,757 |
| 19 | 48,325 | 40,011 | 31,130 | 10,674 | 15,308 | 3,025 | 1,348 | 2,981 | 152,819 |
| 20–24 | 264,824 | 228,163 | 170,060 | 56,270 | 83,261 | 15,183 | 8,237 | 16,681 | 842,755 |
| 25–29 | 294,671 | 249,856 | 178,060 | 57,186 | 97,286 | 15,307 | 11,698 | 17,332 | 921,491 |
| 30–34 | 294,288 | 248,682 | 176,360 | 58,292 | 101,880 | 15,487 | 11,609 | 17,499 | 924,243 |
| 35–39 | 266,090 | 218,478 | 162,839 | 53,275 | 89,517 | 15,220 | 9,607 | 15,785 | 830,943 |
| 40–44 | 256,481 | 207,840 | 165,441 | 52,716 | 85,104 | 15,736 | 8,377 | 14,095 | 805,939 |
| 45–49 | 263,436 | 217,051 | 172,206 | 58,353 | 88,769 | 18,012 | 8,459 | 13,730 | 840,186 |
| 50–54 | 247,863 | 198,491 | 159,538 | 56,814 | 82,235 | 17,911 | 7,519 | 12,281 | 782,812 |

Reference: Australia Bureau of Statistics, June 2017.

**Supplementary Table 6 Index of Relative Socioeconomic Disadvantage**

|  | **Number of person** | **Proportion** |
| --- | --- | --- |
| First quintile | 4,305.1 | 0.1786 |
| Second quintile | 4,835.8 | 0.2006 |
| Third quintile | 4,967.7 | 0.2061 |
| Fourth quintile | 4,987.7 | 0.2069 |
| Fifth quintile | 5,005.9 | 0.2077 |

Reference: National Health Survey: first results, 2017-18, Australia Bureau of Statistics

**Supplementary Table 7 History of smoking and diabetes**

| **Age group** | **Current smoking (%)** | **Ex-smoking (%)** | **Diabetes history (%)** |
| --- | --- | --- | --- |
| 18-24 | 13.9 | 8.5 | 0.6 |
| 25-34 | 14.7 | 20.2 | 0.8 |
| 35-44 | 16.0 | 27.6 | 2.4 |
| 45-54 | 16.9 | 31.5 | 4.5 |
| 55-64 | 15.2 | 37.8 | 10.3 |
| 65-74 | 7.0 | 44.5 | 15.4 |
| 75-84 | 5.1 | 43.5 | 19.6 |
| >85 | 1.7 | 38.3 | 15.4 |

Reference: National Health Survey: first results, 2017-18, Australia Bureau of Statistics

**Supplementary Table 8 Level of systolic blood pressure by age**

|  | Proportion (%) | | | | | | | |
| --- | --- | --- | --- | --- | --- | --- | --- | --- |
| **SBP** | **18–24** | **25–34** | **35–44** | **45–54** | **55–64** | **65–74** | **75–84** | **85 years and over** |
| **<100** | 19.3 | 22.7 | 17.2 | 8.8 | 4.4 | 2.7 | 1.8 | 1.7 |
| **100-110** | 40.2 | 34.8 | 28.0 | 21.7 | 13.6 | 6.6 | 4.4 | 3.2 |
| **110-120** | 28.1 | 24.4 | 25.9 | 20.3 | 18.0 | 12.1 | 9.7 | 7.4 |
| **120-130** | 10.2 | 12.2 | 18.1 | 24.7 | 23.8 | 24.9 | 19.1 | 19.1 |
| **130-140** | 1.7 | 3.7 | 7.1 | 13.7 | 15.8 | 18.6 | 20.4 | 21.6 |
| **140-150** | 0.0 | 1.9 | 2.7 | 5.3 | 13.4 | 15.1 | 18.6 | 17.4 |
| **150-160** | 0.0 | 0.1 | 1.0 | 3.1 | 5.8 | 11.3 | 13.1 | 4.2 |
| **160-170** | 0.0 | 0.2 | 0.0 | 1.8 | 2.2 | 5.0 | 7.3 | 14.5 |
| **>170** | 0.0 | 0.0 | 0.0 | 0.6 | 3.0 | 3.7 | 5.6 | 10.9 |

Reference: National Health Survey: first results, 2017-18, Australia Bureau of Statistics

**Supplementary Table 9 Systolic blood pressure increases by age**

| **Age** | **SBP** | **Increase (mmHg)** |
| --- | --- | --- |
| 16.0 | 118.0 |  |
| 20.0 | 120.0 | 2.0 |
| 30.0 | 124.0 | 4.0 |
| 40.0 | 133.0 | 9.0 |
| 50.0 | 138.0 | 5.0 |
| 60.0 | 154.0 | 16.0 |
| >70 | 165.0 | 11.0 |

Reference: Kotchen JM, McKean HE, Kotchen TA. Blood pressure trends with aging. Hypertension. 1982 Sep-Oct;4(5 Pt 2):III128-34.

**Supplementary Table 10 Level of total cholesterol and high-density lipid by age**

| **TC (<5.5)** | **18–24** | **25–34** | **35–44** | **45–54** | **55–64** | **65–74** | **>75** |
| --- | --- | --- | --- | --- | --- | --- | --- |
| Normal | 87.7 | 72.8 | 65.0 | 54.5 | 51.8 | 62.4 | 73.8 |
| Abnormal | 12.3 | 27.2 | 35.0 | 45.5 | 48.2 | 37.6 | 26.2 |
|  |  |  |  |  |  |  |  |
| **HDL (>1.3)** | **18–24** | **25–34** | **35–44** | **45–54** | **55–64** | **65–74** | **>75** |
| Normal | 76.7 | 72.9 | 74 | 75.8 | 78.1 | 77.2 | 76.7 |
| Abnormal | 23.3 | 27.1 | 26 | 24.2 | 21.9 | 22.8 | 23.3 |

TC: total cholesterol; HDL: high-density lipoprotein

Reference: National Health Survey: first results, 2017-18, Australia Bureau of Statistics

**Supplementary Table 11. Extreme value analysis by assuming a 0% adherence rate after one year of the intervention**

|  | **Lifestyle modification** | **Usual care** | **Difference** | **ICER** |
| --- | --- | --- | --- | --- |
| **Total cost** | $6,964 | $6,668 | $296 | - |
| **QALY** | 18.063 | 18.058 | 0.005 | $58,672/QALY |
| **LY** | 23.076 | 23.072 | 0.005 | $62,017/LY |
| Cost of intervention | $335.6 | $0 | $335.6 |  |
| Cost of CVD-related hospitalisation | $929 | $934 | -$5 |  |
| Cost of CVD management | $5,700 | $5,734 | -$34 |  |

QALY: quality-adjusted life year; LY: life year; ICER: incremental cost-effectiveness ratio; CVD: cardiovascular disease;
